# Angiosarcoma of the Scalp: Metastatic Pulmonary Cystic Lesions Initially Misinterpreted as Benign Findings on 18F-FDG PET/CT

**DOI:** 10.3390/diagnostics6010001

**Published:** 2015-12-22

**Authors:** Kim Francis Andersen, Elisabeth Albrecht-Beste, Anne Kiil Berthelsen, Annika Loft

**Affiliations:** Department of Clinical Physiology, Nuclear Medicine & PET, Rigshospitalet, Copenhagen University Hospital, Blegdamsvej 9—PET 3982, DK-2100 Copenhagen, Denmark; Elisabeth.Albrecht-Beste@regionh.dk (E.A.-B.); anne.kiil.berthelsen@regionh.dk (A.K.B.); annika.loft.jakobsen@regionh.dk (A.L.)

**Keywords:** angiosarcoma, pulmonary metastasis, FDG PET

## Abstract

Angiosarcomas are rare and only represent about 2% of all soft tissue sarcomas. They arise from vascular or lymphatic endothelial cells and are most commonly located in the heart, liver, breast, and skin. Cutaneous angiosarcoma of the scalp is highly malignant and with dismal prognosis. Reported five-year survival is <30%. The mainstay of treatment is surgical resection and adjuvant radiation therapy, but failure rates following local therapy are high. Cutaneous angiosarcoma of the scalp has a predilection for pulmonary metastases with a variety of morphologic patterns on imaging. Metastatic disease in terms of pulmonary thin-walled, cystic lesions, may not be hypermetabolic on ^18^F-FDG PET and, as such, could be misinterpreted as benign findings. We present a case demonstrating the diagnostic uncertainty and delay in an elderly male with angiosarcoma of the scalp presenting with metastatic lung lesions following failure of local therapy.

**Figure 1:**
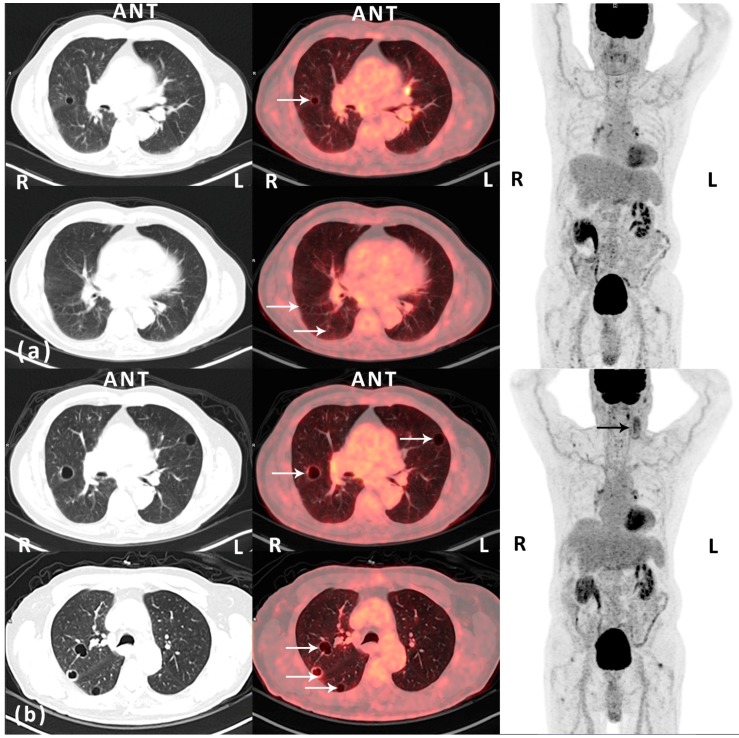
(**a**) Post-operative (12 months after surgery) routine ^18^F-FDG PET/CT scan (Siemens Biograph mCT-S 64, Siemens Medical Solutions, Knoxville, TN, USA) performed approximately 1 h after an intravenously injected ^18^F-FDG dose of 4 MBq/kg body weight—left and middle: CT (lung window) and fused ^18^F-FDG PET/CT, transaxial view; right: ^18^F-FDG PET, maximal intensity projection. An 85-year old man with a history of well-differentiated T1 bladder cancer with no signs of relapse on follow-up. The patient presented with a rapidly growing nodular, vascular tumor (7 × 7 cm) in the left parietal region of the scalp. Histopathology prior to surgery was inconclusive. Macroscopically radical resection of the tumor was performed. However, microscopically, the deep surgical margin was reported as marginal. Histopathology demonstrated a hemorrhagic and necrotic tumor with infiltration of the dermis and subcutaneous tissue. In vital areas of the tumor there were vascular spaces with atypical endothelium positive for CD31, CD34, and podoplanin. Ki-67 index in was approximately 30%. The diagnosis was angiosarcoma. The patient was referred to a specialized sarcoma center for adjuvant therapy and follow-up. The first post-operative computed tomography (CT) of the thorax and abdomen (1 month after surgery) demonstrated multiple cysts in the kidneys and the liver. Also, there was a lesion in the spleen from which ultrasonography (US)-guided biopsy came out with hemangioma. No pulmonary lesions were identified. Adjuvant curative intended intensity-modulated radiation therapy (IMRT) with RapidArc (TD 66Gy, 2Gy/f, 5f/w) was finalized without significant complications 4 months post-operatively. At clinical follow-up, one year after surgery, the patient presented with acute swelling on the left side of the neck. Metastatic disease was suspected. However, US-guided fine needle aspiration cytology from a tumor adjacent to the left parotic gland demonstrated inflammation. This was supported by findings on a fluorine-18 fluoro-2-deoxy-d-glucose positron emission tomography (^18^F-FDG PET)/CT, which also demonstrated several thin-walled pulmonary cystic lesions on the right side with a diameter of up to 1.1 cm and with no FDG-avidity (white arrows, HU -989; maximal standardized uptake value (SUV_max_) of 0.94 g/mL). The lesions were reported as benign. The opinion of the multidisciplinary tumor board was to recommend the patient routine clinical and imaging follow-up; (**b**) Follow-up (18 months after surgery) ^18^F-FDG PET/CT scan (the same PET/CT scanner and acquisition of data as previously described)—left and middle: CT (lung window) and fused ^18^F-FDG PET/CT, transaxial view; right: ^18^F-FDG PET, maximal intensity projection. Prior to the scan blood samples showed microcytic, hypochromic anemia due to iron deficiency. The patient had initiated anticoagulant therapy due to an episode of deep vein thrombosis in the right leg and bilateral pulmonary embolism. The patient reported a decline in general well-being and increased tiredness. Also, the swelling on the left side of the neck persisted. US was performed and now identified two cystic-solid tumors, from which fine needle aspirations were performed. Cytology was inconclusive. However, ^18^F-FDG PET/CT demonstrated morphologically and metabolically inhomogeneous lesions in the same region, which progressed when compared to the ^18^F-FDG PET/CT 6 months earlier (black arrow). Also, progressive disease in terms of increased size (largest diameter 2.1 cm) and number of previously reported cystic lesions in both lungs was seen, now with involvement of all lobes (white arrows). The peripheral part of some of the lesions was now seen with pathologically slightly increased FDG-uptake, corresponding to the thin, partly irregular wall of the cystic lesions (SUV_max_ 2.18 g/mL). The lesions on the neck and in the lungs were reported as suspected metastatic disease. Additionally, as seen on the MIP images, there were some FDG-avid lesions located in the mediastinum/hilum of the lungs and in soft tissue adjacent to joints—these lesions were stable over time and reported as non-malignant. The recommendation of the multidisciplinary tumor board was to perform an excisional biopsy of the most superficial lesion (lymph node) on the neck. Histopathology was consistent with metastatic angiosarcoma. Consequently, the pulmonary lesions were considered as metastases from the primary angiosarcoma located on the scalp. The patient was referred to palliative chemotherapy with paclitaxel. This case illustrates pit-falls on imaging in a patient with angiosarcoma of the scalp with metastatic pulmonary lesions on follow-up ^18^F-FDG PET/CT scans, which initially was misinterpreted as benign, partly due to the lack of pathologically FDG uptake on the PET scan. Angiosarcomas are rare, only representing about 2% of all soft tissue sarcomas [1,2]. They arise from vascular or lymphatic endothelial cells and are most commonly located in the heart, liver, breast, and skin. Cutaneous angiosarcoma of the scalp is most often seen in elderly men, is highly malignant and with dismal prognosis. Reported five-year survival is <30% [3,4,5], but it is unclear which prognostic factors are of the most importance. Tumor size > 5 cm, metastasis at the time of diagnosis, high tumor grade and tumor necrosis, absence of surgical resection, old age, poor patient performance status, visceral/deep tissue locations of the disease, and prior radiation have all been suggested as factors for poor outcome. The mainstay of treatment is surgical resection and adjuvant radiation therapy, but failure rates following local therapy are high. Cutaneous angiosarcoma of the scalp has a predilection for pulmonary metastases with a variety of morphologic patterns on imaging [6,7], with reports of multiple solid nodular lesions, ground glass attenuation, septal thickening from lymphangetic spread, and thin-walled cystic lesions with possible air–fluid levels within the cysts as an indication of hemorrhage. Cystic or bullous lung changes in a patient with known sarcoma are considered a sign heralding metastatic disease. Several mechanisms have been suggested for the genesis of these metastatic pulmonary cystic lesions [7,8]: (1) excavation of solid nodular lesions; (2) infiltration of tumor cells into the walls of preexisting benign bullous changes; (3) infiltration of tumor cells into the walls of air sacs formed by distension of small airways through the ball-valve effect of the tumor; and (4) tumor cell proliferation to form characteristic blood-filled cystic spaces. Literature on ^18^F-FDG PET imaging of lung manifestations of metastatic angiosarcoma of the scalp is extremely sparse. A single case-report suggests that metastatic disease in terms of pulmonary thin-walled, cystic lesions may not be hypermetabolic on ^18^F-FDG PET and, as such, could be misinterpreted as benign findings [8]. This is coherent with our findings, which, due to the thin solid component of the cystic lesions and the limited resolution of the PET scanner, demonstrated a low SUV_max_, even though a relatively high Ki-67 index in the primary tumor should indicate the opposite. This case also demonstrates the diagnostic uncertainty and delay in an elderly male with angiosarcoma of the scalp with metastatic pulmonary lesions following failure of local therapy, which all probably affects the generally poor outcome in these patients. However, continuous follow-up imaging demonstrated progressive metastatic disease. We believe that this case-report emphasizes the importance of knowing the pathogenesis behind pulmonary metastasis and the variety of presentation of this manifestation on different imaging modalities in a rare disease such as angiosarcoma.
